# Selective Heterogeneity in Exoprotease Production by *Bacillus subtilis*

**DOI:** 10.1371/journal.pone.0038574

**Published:** 2012-06-20

**Authors:** Fordyce A. Davidson, Chung Seon-Yi, Nicola R. Stanley-Wall

**Affiliations:** 1 Division of Mathematics, University of Dundee, Dundee, United Kingdom; 2 Molecular Microbiology, College of Life Sciences, University of Dundee, Dundee, United Kingdom; University of Nottingham, United Kingdom

## Abstract

Bacteria have elaborate signalling mechanisms to ensure a behavioural response that is most likely to enhance survival in a changing environment. It is becoming increasingly apparent that as part of this response, bacteria are capable of cell differentiation and can generate multiple, mutually exclusive co-existing cell states. These cell states are often associated with multicellular processes that bring benefit to the community as a whole but which may be, paradoxically, disadvantageous to an individual subpopulation. How this process of cell differentiation is controlled is intriguing and remains a largely open question. In this paper, we consider an important aspect of cell differentiation that is known to occur in the Gram-positive bacterium *Bacillus subtilis*: we investigate the role of two master regulators DegU and Spo0A in the control of extra-cellular protease production. Recent work in this area focussed the on role of DegU in this process and suggested that transient effects in protein production were the drivers of cell-response heterogeneity. Here, using a combination of mathematical modelling, analysis and stochastic simulations, we provide a complementary analysis of this regulatory system that investigates the roles of both DegU and Spo0A in extra-cellular protease production. In doing so, we present a mechanism for bimodality, or system heterogeneity, without the need for a bistable switch in the underlying regulatory network. Moreover, our analysis leads us to conclude that this heterogeneity is in fact a persistent, stable feature. Our results suggest that system response is divided into three zones: low and high signal levels induce a unimodal or undifferentiated response from the cell population with all cells OFF and ON, respectively for exoprotease production. However, for intermediate levels of signal, a heterogeneous response is predicted with a spread of activity levels, representing typical “bet-hedging” behaviour.

## Introduction

Bacteria are capable of “multicellular” behaviours that benefit the bacterial community as a whole [1]–[4]. How bacteria integrate several mutually exclusive cell states within the population is intriguing and understanding this phenomenon is essential to determining, for example, why there are antibiotic resistant persistor cells [5], why subpopulations of cells have different fates in a differentiating species, such as sporulating species [6], and even why there is variability in motile and chemosensory behaviour in swimming species [7]. Studying the origins and consequences of this heterogeneity within the population is therefore central to our ability to understand, control and exploit these ubiquitous organisms. To address the question of how heterogeneity is manifest in a bacterial population, we present a mathematical analysis of the mechanisms underpinning the heterogeneity of extracellular protease (exoprotease) production in the model Gram-positive bacterium *Bacillus subtilis*.

The production of extracellular proteases (exoproteases) functions to provide nutrients for the cells to grow and divide [8]. Furthermore, a significant increase in the production of exoproteases has been correlated with an inhibition of biofilm formation [9]. This raises the possibility that the exoproteases potentially function to reverse biofilm formation by degrading the protein components of the extracellular matrix that binds the cells within the biofilm [9]. This could allow for a return of the cells to a free-swimming state. In passing, we note that it is known that multicellularity in *B. subtilis* not only encompasses the production of exoproteases [8] but also swarming motility [10] genetic competence [11]–[13], sporulation [14], [15] and biofilm formation [16], [17].

*B. subtilis* exoprotease production is controlled by the activation of two regulators; namely Spo0A and DegU [18], [19]. Entry to (or indeed exit from) different cell states is dependent in part on the level of phosphorylated DegU within the cell [9], [20], [21]. The level of phosphorylated DegU is controlled by its cognate sensor kinase, DegS [20]. The DegS-DegU two-component regulatory system regulates at least five multicellular processes in *B. subtilis*: biofilm formation; genetic competence; swarming motility; polyglutamic acid production and; protease production [19]. In the absence of an appropriate environmental signal, DegS is at a low level and unphosphorylated and the level of phosphorylated DegU is also low, as its production is regulated by a positive feedback loop [8], [22]. The production of the extracellular proteases occurs when the DegS-DegU system is stimulated [9], [20], [21]. It is not known exactly what the environmental stimulus perceived by DegS is, but changes in osmolarity [23], carbon levels or amino acid starvation [24] have all been hypothesized. Moreover, how such two-component systems integrate multiple cell states into the population is unclear. However, the detection of such a signal is likely to be subject to variation and therefore likely to induce changes in the phosphorylation status of DegU at the individual cell level. The other key regulator under consideration here is Spo0A, which is activated by phosphorylation mediated by a phosphorelay system that is, in part, stimulated by starvation [14], [15]. Spo0A
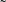
P regulates the expression of approximately 120 genes according to the specific affinity of the promoter element for Spo0A
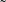
P [25]–[27]. This allows the activation of different subgroups of genes at different levels of Spo0A
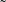
P [26], [28]. Spo0A
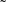
P is most commonly associated with sporulation. However, the main role of Spo0A
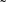
P during the activation of exoprotease production is to facilitate transcription from the *bpr* (bacillopeptidase) and *aprE* (subtilisin) genes. Spo0A
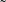
P achieves this by removing the direct inhibition of transcription exerted by two repressor proteins called SinR and AbrB [21], [27], [29].

Mathematical modelling of gene regulatory networks in bacteria is an area of intense research interest. Of particular relevance to the work presented here, genetic switches have attracted much attention [30]–[33]. Both deterministic and stochastic approaches have been used to explain the complex (non-linear) interplay between multi-component systems and the effects of intrinsic and extrinsic noise in determining cell fate. Again of relevance to the work here, the model bacterium *Bacillus subtilis* has attracted considerable attention (for an excellent overview, see [34]). The master regulator Spo0A discussed here also mediates many other pathways, in particular, sporulation (see e.g. [35], [36]). Jabbari et al. [37] construct deterministic models with which they explore the interplay between quorum sensing, nutrient levels, DNA damage and competence in determining what induces the irreversible decision to form spores. Further details of the role of quorum sensing in the sporulation route is discussed in e.g. [38]. There, using a combination of deterministic and stochastic models, it is demonstrated how the phosphorelay may be able to integrate environmental signals to compute “food per cell” and use this measure a determining factor in the decision to sporulate.

It is our overall goal to understand how DegU regulates multicellular processes and in particular how its regulatory network intersects with other genetically separate regulatory pathways. In a step towards this goal, in this paper we present a complementary mathematical analysis of recent work presented in [8] where Veening et al. used a combination of experimental methods and mathematical modelling to investigate how the master genetic regulators, Spo0A and DegU, interact to control cell fate in *B. subtilis*. In particular, they demonstrated that activation of both gene regulators was required to initiate exoprotease production and thus this pathway interaction was described as AND logical. The main conclusion of [8] was that stochastic effects in gene regulation and transcription governed *temporal heterogeneity* in the expression of the resultant protein and thus the heterogeneous cell activity observed in the laboratory. Here we show that an alternative analysis of the underlying model structure reveals that heterogeneity in protein levels is not simply a transient feature of this regulatory network, but rather a persistent, steady state property of the system. Moreover, we discuss how this heterogeneity in system response is mediated by the level of the input signal and further investigate the AND logic discussed above. We call this system response *selective heterogeneity* to differentiate it from the transient, *temporal heterogeneity* discussed previously. Essentially, we show that the shape of the steady state system signal-response curves induces a lensing of stochastic effects: the system is differentially sensitive to changes in input signal and different components within the system are more or less sensitive to changes to those signals. In the deterministic limit, the model predicts that bimodal cell distributions (in our case DegU ON, exoprotease OFF and DegU ON, exoprotease ON) result without the system exhibiting classic bistable kinetics. Stochastic effects smear this deterministic limit to yield a heterogeneous population of cells all of which are DegU ON but for which the exoprotease status can vary by up to 3-fold. This long-term, stable heterogeneity in cell type may have particular relevance to understanding the development of biofilms within which differentiated cell subpopulations cooperate over an extended time period [39].

## Methods

### Model Formulation

**Figure 1:**
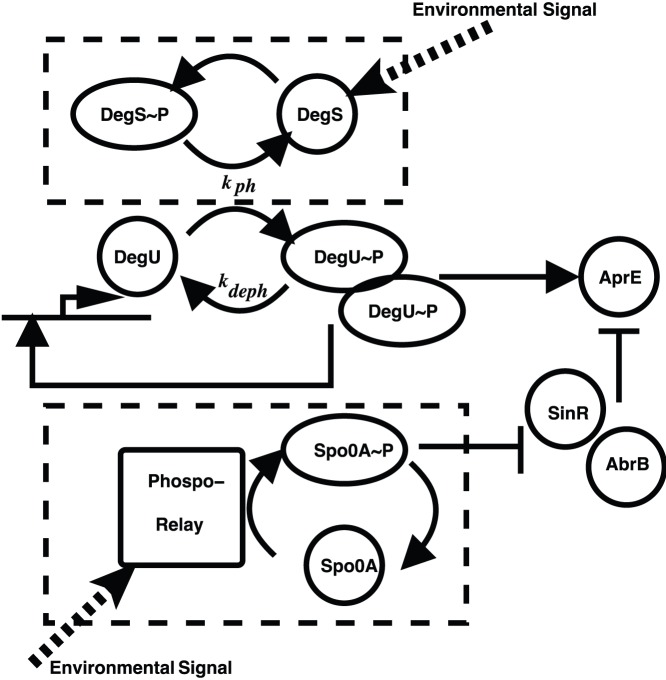
Schematic of the DegU - Spo0A intersecting control networks. The interaction of the main components of the model are illustrated. Interactions enclosed in dashed boxes are not explicitly considered. See text for further explanation.

The model presented in [8] is centred on the dynamics of DegU auto-regulation. A schematic of the components and how they interact is given in Figure 1. As illustrated, the main output of the system is the level of exoprotease expression (number of AprE molecules per cell). This system output is governed by two independent networks: (i) the DegU/DegU
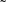
P module and (ii) the SinR/AbrB module which is regulated by Spo0A. These modules are themselves activated by environmental signals. How the cell perceives and processes environmental signals is highly complex and involves both the role of DegS, the sensor histidine kinase that phosphorylates and hence activates DegU and a complex phosphorelay that activates Spo0A. It is our intention to focus on the downstream processes from this signal transduction machinery. Hence, in the model, Spo0A variations will be considered to implicitly affect the levels of AbrB and SinR as detailed below. The role of DegS will be encapsulated in an “effective” phosphorylation rate 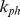 and hence 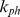 can be thought of as the signal response parameter and will be referred to as the **signal parameter/signal strength** for short. We are principally interested in studying system output in response to changes in this signal parameter.

The model comprises four core components: mRNA *degU*, the DegU protein, phosphorylated DegU (DegU
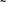
P ) and a dimer of phosphorylated DegU. In the model, the number of molecules per cell of these components is denoted here by 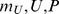 and 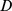, respectively. The dimer of DegU
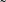
P controls (in part) the downstream production of exoproteases: the variables in that component of the model are the mRNA associated with the exoproteases and the proteins themselves, denoted by 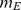 and 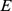, respectively. Following standard mass action arguments (that the rate of reaction is proportional to the product of the concentration of the reactants), the dynamic interaction of these variables can be modelled using the following system of ordinary differential equations, which describes the net rate of change in the (average) number of molecules per cell. Each equation can be read as

net change over a small time interval  =  production − loss.

Production is via transcription, translation or species-change, e.g. phosphorylation or dimerisation. Loss is via degradation or species-change. The system of equations reads as follows:
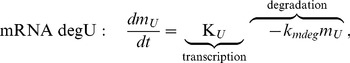
(1)

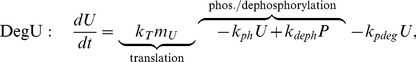
(2)

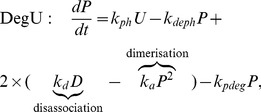
(3)

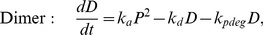
(4)

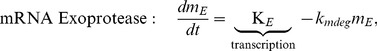
(5)

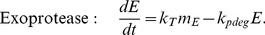
(6)


(The factor 2 in the final term in (3) is included to ensure the correct mass conservation law arising from the dimerization of monomers of 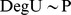: all other processes excluded, 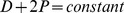 - see e.g. [40]. We note that this factor is missing in the formulation that appears in [8], but do not view this as critical to the qualitative properties of the results presented there (or here).

The term 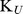 represents the transcription flux from the *degU* promoter. It can be considered to comprise two processes: (i) a basal level of transcription that occurs in the absence of stimulation from the phosphorylated dimer of DegU and (ii) transcription in response to DegU
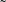
P dimer stimulation. This flux can therefore be written as
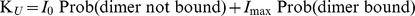


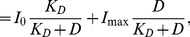
(7)where 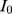 and 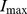 represent the maximal basal and stimulated flux, respectively and 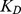 is the half-maximal binding constant (i.e. the level of 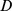 at which each of these processes are at their half-maximal rates). The underlying assumption that leads to the standard representation of transcription (7) is that the association and dissociation of the dimer of DegU
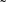
P with the promoter site is relatively fast.

The transcription flux from the DegU
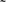
P -dependent exoprotease promoter elements (such as the *aprE* and *bpr* promoter regions [41]–[44]) is more complex. Transcription from these promoters is known to be activated by the DegU
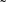
P homodimer, denoted here by 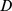, but it is also repressed by the regulators SinR and AbrB. Following Veening et al., we will focus on the control of *aprE* but refer to this hereafter as exoprotease to emphasise the generic element of this control mechanism. It is assumed that (i) there is a basal level of transcription, (ii) the promoter site is capable of binding two dimers, which act accumulatively to promote transcription, (iii) AbrB interferes with the action of these dimers by competing for the binding site and (iv) the binding of SinR takes place upstream of the promotor site and thus its action is to regulate transcription irrespective of the dimer level. Hence, an expression for the transcription flux is as follows:
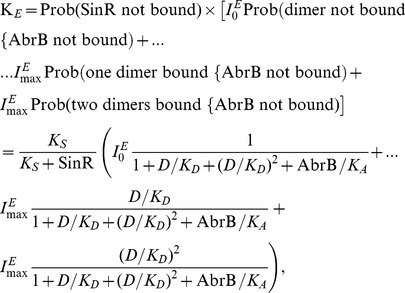
(8)


where, as above, 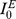 and 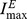 represent the maximal basal and stimulated flux, respectively, 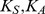 and 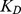 are the half-maximal binding constants for SinR, AbrB and the DegU
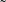
P dimer, respectively. No explicit account of the dynamics of SinR and AbrB is taken at this point, they are simply set as constants. Later, we investigate the consequences of varying these values thus mimicking differential signalling through the Spo0A signalling pathway and explicitly demonstrate the AND logic of this regulatory system.

The meaning of the parameters and their values (used throughout unless otherwise stated) are given in Table 1. These values are either taken directly from [8], the accompanying Information S1 or the accompanying numerical code. An extended discussion of how these values are estimated is provided in the Supplementary Information for [8]. In short, many of the values are estimates based on known, similar reaction rates and/or are computed from basic observations of the reaction scheme under investigation and physical principles, in particular time scales. It is noted there and confirmed here that the system is not in general qualitatively sensitive to small changes in the parameter values. Indeed, as discussed below, certain key features are insensitive even to changes of order. Some of the values are stated in terms of number of molecules or “copy number” 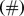. A copy number of approximately 602 corresponds to a concentration of approximately 1
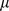
M/l [8]. Note that systems of the type (1)–(6) are more normally couched in terms of concentrations and hence, naturally, the variables are real-valued, continuous functions of time. For ease of comparison with stochastic simulations that will be discussed later, the system is written in terms of (average) numbers of molecules per cell and hence these can be again viewed as real-valued continuous functions. (Of course, the stochastic simulations return integer-valued outputs.) A suitable scaling of the rate constants given in Table 1 maps one formulation to the other whilst the dynamics remain unaltered.

**Table 1:** Parameter Values.

parameters	values	description of constants
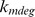	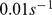	mRNA degradation
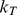	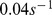	translation per Mrna
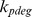	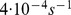	mRNA degradation
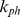	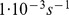	DegU phosphorylation
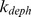	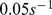	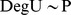 dephosphorylation
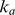	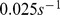	dimer of 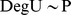 association
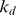	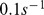	dimer of 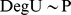 dissociation
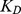	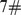 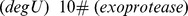	equim. of 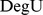 dimer binding to promoter
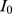	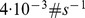	transcription of *degU* from inactivated promoter
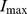	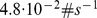	maximal transcription of *degU* from activated promoter
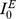	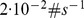	transcription of *exoprotease* from inactivated promoter
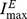	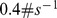	maximal transcription of *exoprotease* from activated promoter
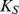	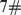	dissociation of SinR binding to promoter
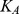	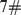	dissociation of AbrB binding to promoter

Parameter descriptions and values from [8].

### Numerical Integration

#### System dynamics

The dynamics of system (1)–(6) can be determined by numerical integration using any reasonable ode solver. We chose to use the MATLAB ode45 programme, which employs a Runge-Kutta 4-5 predictor-corrector algorithm. Standard checks were made to ensure that decreasing error tolerances did not alter the qualitative or quantitative properties of the solution.

#### Steady state analysis

To complement the mathematical analysis, the behaviour and stability of the steady state response curves of the system (1)–(6) were determined using the well-known numerical continuation package AUTO. We used the lasted version of this package AUTO 07p for the simulations discussed in the Results section. Graphical output was generated using MATLAB R2010b.

#### Modelling noise

If the number of interacting molecules is very low, then it is possible that noise plays a significant role in the system dynamics. Therefore it is appropriate to consider an alternative modelling approach that incorporates stochastic effects in the reactions. This can be done using the Gillespie Stochastic Simulation Algorithm (GSSA) [45] to compute typical solution paths. (See for example [46] and [47] for a very clear introduction and comprehensive overview, respectively.) In short, this method replicates the statistics of random interactions between reactant molecules within a given volume. Stochastic effects induced by low copy numbers are often referred to as *intrinsic* noise. To generate the results discussed below, we used the GSSA approach without deviation and implemented it using the systems biology freeware package Dizzy [48]. Each run of the algorithm can be viewed as the temporal evolution of the transcription network within a single cell. Data from multiple runs can therefore be directly compared to data produced by flow cytometry techniques in which the level of transcription in single cells is associated with the level of fluorescence generated by the green fluorescent protein, whose production is driven by the heterologous promoter region under consideration (see [49]). In this paper, we report data produced from 1000 stochastic simulations in each case. This is equivalent to presenting the data from 1000 individual cells. The model considers output from the network over a 17 hour period. (This final time represents e.g. typical overnight culture of biofilms. In all the simulations we discuss below, the system is in steady state by this time and hence further time integration does not yield any more information.) The programme Dizzy is not capable of storing data from multiple simulations (other than in averaged format) and therefore a short shell script was written to perform the loop and store the data from each run separately. In the case where different, randomly assigned values of the phosphorylation rate were required, these were first created using the pseudo-random number generator in MATLAB R2010b and then loaded into the Dizzy equation files, again using a short shell script. Data from each run were stored in CSV format and a MATLAB script was used to collate the data and generate histograms.

## Results

### System Dynamics

**Figure 2:**
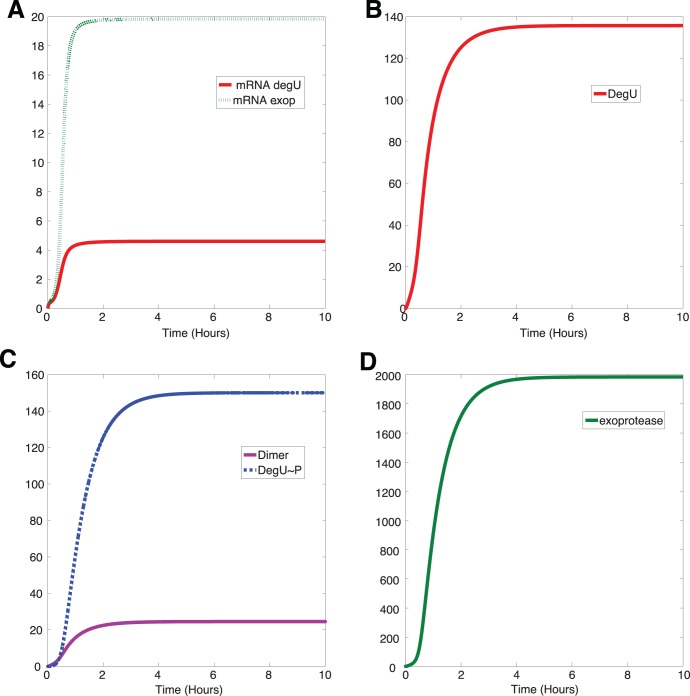
Dynamics of the DegU and exoprotease systems. Temporal evolution of the DegU - exoprotease system as modelled by equations (1)–(6). The levels of (A) mRNA, (B) DegU, (C) DegU
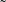
P and dimer of DegU
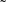
P and (D) exoprotease. All parameters as in Table 1.

The time evolution of copy numbers as predicted by the model is shown in Figure 2. For ease of comparison, the initial level of all reactants was set to zero, representing the case where the cells can be considered to be in an OFF state. All reactants increase monotonically and settle to a steady state value after approximately 5 hours. It is important to note that different choices of the rate of phosphorylation of DegU, 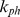, and initial protein levels resulted in transient times of the same order, hence underpinning the robustness of the model predictions discussed below. Indeed, setting all reactants to zero at the start of the simulation represents an extreme case that will generate the long transient times; other choices of initial data produced shorter transient times (see Fig. S1). Moreover, this transient *biochemical reaction time-scale* is considerably shorter than e.g. time to the steady-state growth phase in liquid culture or typical studies of biofilm development. Comparing Figures 2A and 2D provides a scale for the range of copy numbers resulting from the system. We note that it is currently impossible to experimentally distinguish between the different components of the total pool of DegU. This is due in part to the instability of the phosphate moiety on the aspartic acid at position 56 on DegU during *in vitro* manipulation. However, the model predicts that the temporal evolution of each of the DegU components (shown in Figure 2B and 2C) follows the same profile. Moreover, and of greater pertinence to the work discussed here, in steady state the level of DegU in the cell dominates and thus the steady state response curves for DegU and the total DegU pool are indistinguishable, for signal parameter values in the range under consideration (see Fig. S6). Finally, it is known that the unphosphorylated from of DegU controls competence [11]–[13] and therefore tracking the evolution this component is of importance in its own right. Hence, the figures below will only display output for DegU.

### The Steady State Framework

Given the relatively short transient illustrated in Figure 2, a framework with which to better understand the longer term system response to different signal strengths is formed by considering the steady states of (1)–(6). This is done by setting the left hand sides to zero in each of the equations and results in a system of six algebraic, nonlinear equations, the solutions of which provide the steady states. System properties can be determined by investigating how these steady states change with parameters, in particular the signal surrogate, 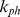. Note that systems with non-linear positive feedback mechanisms as is the case here, have the necessary properties to produce a wide range of complex behaviours including bistability. We now discuss system behaviour first by considering numerical computations and then via a detailed analysis of a related but simpler system.

**Figure 3:**
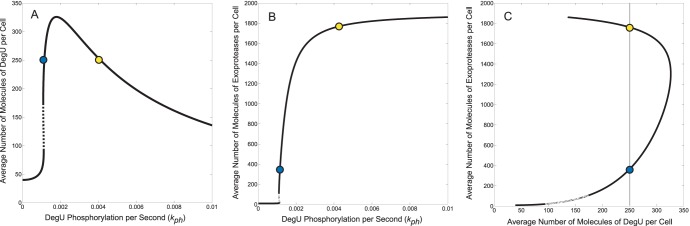
The steady state values of DegU and exoprotease as functions of the phosphorylation rate 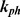 as predicted by the full system. Steady state solutions for (A) 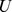 (DegU) and (B) 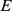 (exoprotease) of system (1)–(6) as functions of 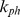. (C) Plot of DegU versus the level of exoprotease (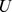 versus 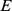). Solid lines represents stable solutions, dashed lines represent unstable solutions and a region of bistability. The blue and yellow dots individually indicate system output for two different values of 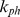. They are provided for ease of comparison between the graphs (see text). All parameter values from Table 1.

Using the default parameter set given in Table 1, numerical solution (continuation) of the steady state equations corresponding to the full system (1)–(6) yields the steady state signal response diagram shown in Figure 3. Two qualitative features are immediately obvious: (i) the system response curve for DegU displays a distinct fold (Figure 3A) and (ii) for signal values 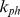 corresponding to the location of this fold, the exoprotease level displays an ultra-sensitive switch. Consequently, for two similar signal values, stable steady states can comprise the same DegU values but very different exoprotease values (compare system output indicated by the blue and yellow dots, see also Figure 3C). So the model predicts that cells could potentially respond bimodally to similar signal values: all cells would be ON for DegU expression but a fraction of cells would be respectively ON and OFF for exoprotease production. Moreover, in the Information S1 it is shown that the relationships shown here hold even if DegU is replaced with the total DegU pool (DegU + DegU
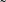
P + dimer), see Figure S6.

**Figure 4:**
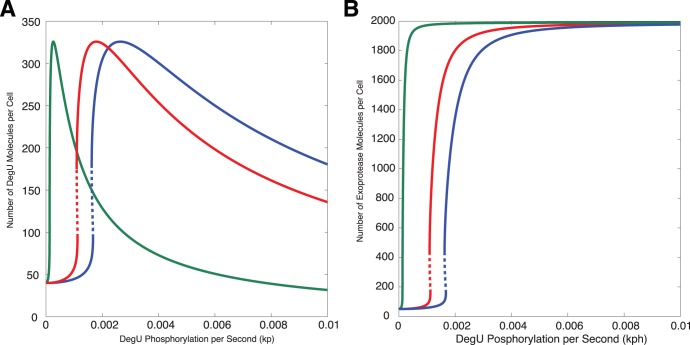
How the steady state curves change with the dephosphorylation rate 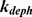. Steady state solutions for (A) 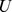 (DegU) and (B) 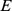 (exoprotease) of the full system (1)–(6) as functions of 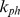. Solid lines represent stable solutions, dashed lines represent unstable solutions and a region of bistability. All parameter values from Table 1 except 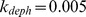 (green); 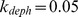 (red); 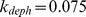 (blue).

The form of these response curves depends on parameter choice. Extensive numerical simulations revealed that both the qualitative and quantitative properties are relatively insensitive to small changes in parameter values. Not surprisingly, the location and height of the fold did change with 2-fold changes in most parameters and the region of bistability (indicated by the dashed lines in Figure 3) could be increased by reducing 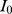 below the default value and eliminated by increasing 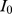. However, and more interestingly, the “width” of the fold (measured here as the distance between the corresponding 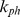 values associated with a value approximately 75% of the maximum DegU level) appeared to be insensitive to 2-fold variations in almost all the system parameters and insensitive to 10-fold changes in some, e.g. 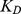, 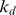 and 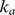. (Of course all features of the DegU response curve are independent of parameters related to the downstream exoprotease component of the model.) It is unclear whether such variations in parameter values are biologically relevant, but it provides a better understanding of the underlying mathematical structure and suggests that the diagrams shown in Figure 3 are qualitatively representative. The exception seems to be variations in 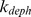 for which the fold height was not affected, but the fold width scaled (non-linearly) with 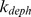. Figure 4 shows system output for different values of 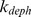. Notice that the corresponding switch in exoprotease level tracks the location of the fold. Hence, the two steady states indicated by the blue and yellow dots in Figure 3 can be associated with signal values 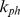 that are relatively close together. This led us to investigate stochastic variations in effective signal values that could exist between cells and/or arise from the heterogeneity of the cell microenvironment. If such a variation exists the question is what would the consequences be? First, however we discuss how a reduced model provides a better understanding of the generic form of the signal response curves and their robustness to parameter variation.

#### A minimal model: generic behaviour and robustness

The superlinear forms of 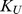 and 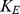 ensure that no useful closed form expressions for the steady states of the system exist: only numerical solutions can be found as detailed above. However, considerable further insight into how the steady state values change with parameter values of the model predictions can be gained by considering an approximate or minimal system as we now discuss. Full details of the derivation and analysis of the minimal system appear in the Information S1, see also Figs. S2, S3, S4, S5, S6.

**Figure 5:**
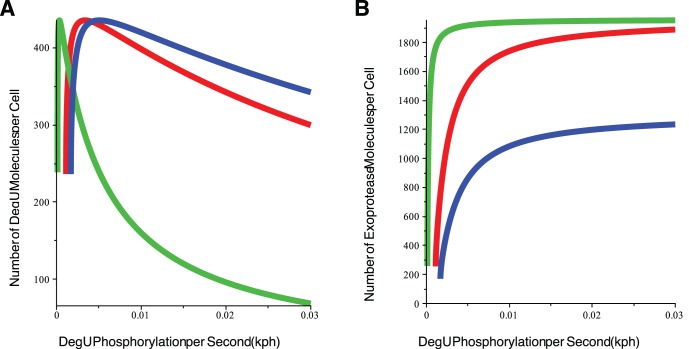
The steady state values of DegU and exoprotease as functions of the phosphorylation rate 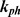 as predicted by the minimal system. Steady state solutions derived using (9). (A) 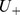 (DegU) and (B) 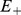 (exoprotease - computed using (S7)–(S9)) as functions of 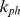. In each figure, all parameter values from Table 1 except 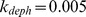 (green); 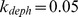 (red); 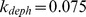 (blue).

In summary, a minimal system is obtained by assuming the processes of phosphorylation and dephosphorylation of DegU and the association and dissociation of the dimer of DegU
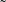
P are fast compared to protein production and degradation. A further approximation is made in which it is assumed that the background transcription rate 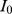 of *degU* is small compared to the maximal rate 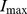. The first set of these assumptions reduces the six algebraic equations associated with steady states of the full model to one cubic equation for the level of DegU:
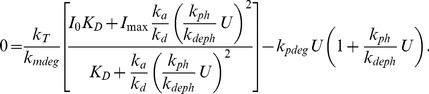
(9)Three slave equations provide the corresponding values for DegU
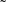
P, the dimer and exoprotease. With 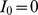, and considering all other parameters fixed, (9) has the trivial solution 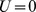 and two positive solutions 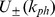. Full details of the behaviour of these solutions, the corresponding values of the other variables and the behaviour as 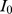 is increased from zero are given in the SI. Figure 5 shows the dependence of the DegU and exoprotease levels as predicted by (9) using solutions 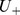 and 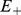. These solutions display the same qualitative response to changing the parameter 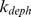 as the full model shown in Figure 4. A distinctive fold when viewed as a function of the phosphorylation rate 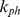 is observed that coincides with an ultra-sensitive switch in exoprotease level. An analysis of this minimal model reveals that the width of the peak is proportional to 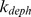. Moreover, although the switch in exoprotease is sharp, the analysis reveals that DegU response curve increases more rapidly with increasing 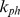 than the exoprotease response level. Hence the latter always straddles the former. This analysis suggests that the features illustrated in Figure 4 are not degenerate and in particular the alignment of the fold with the ultra-sensitive switch that generated the bimodal behaviour predicted by the full model is robust and not a consequence of the specific choice of default parameter values. We now discuss the effects of both intrinsic and extrinsic noise on this key predicted (deterministic) feature of the regulatory system.

### Selective Heterogeneity and Bimodality

In [8], the main focus was on dynamic effects as a source of stochastic heterogeneity in levels of expression. The idea presented there was that, for phosphorylation rates in the vicinity of a region of bistability of DegU, stochastic effects caused some system trajectories to be trapped close to the “wrong” steady state for some time before converging to the appropriate stable state. The conclusion was that this temporal smearing of transcription level between cells induced heterogenous outputs at the population level.

The numerical simulations shown here in Figure 2 predict that the movement of the system to steady state is in fact quite rapid (in the deterministic case at least). Moreover, for the parameter values considered here and in [8], the range of 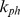 values for which DegU is bistable, is very narrow (see region indicated with dashed line in Figure 3A). This motivated us to consider other possible effects by which heterogeneous expression levels could be induced **at steady state**. In particular, we assessed the effects of low copy numbers (intrinsic noise) and signal heterogeneity as manifest through changes to the phosphorylation rate 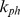 (extrinsic noise) as potential drivers of stochasticity in expression level. These would represent situations where individual cells within a population have small differences in their ability to either detect or respond to environmental signals as perceived by DegS. This situation is particularly relevant in the natural environment or within the confines of structured biofilm communities, where, additionally, the microenvironment is likely to be heterogeneous [39].

#### Intrinsic noise: low copy numbers

**Figure 6:**
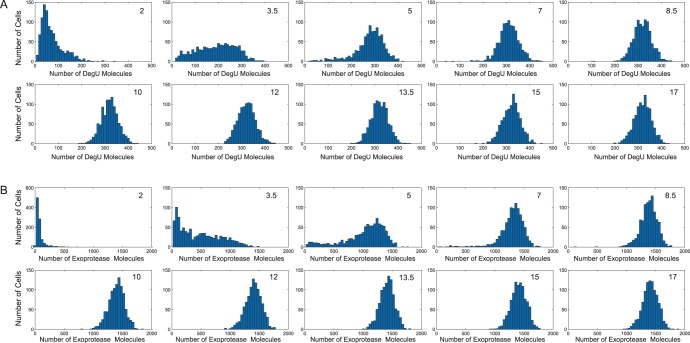
Temporal evolution of protein levels subject to intrinsic noise. Output from system (1)–(6) computed using the Gillespie SSA. Histograms show the levels of (A) DegU and (B) exoprotease collated from 1000 simulations at 10 time points. Each simulation can be considered to be equivalent to the temporal evolution of transcription in a single cell. Note that distributions for both DegU and exoprotease display no significant further change after approximately 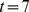 hrs. All parameter values from Table 1 except 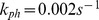. Initial protein values set to zero.

**Figure 7:**
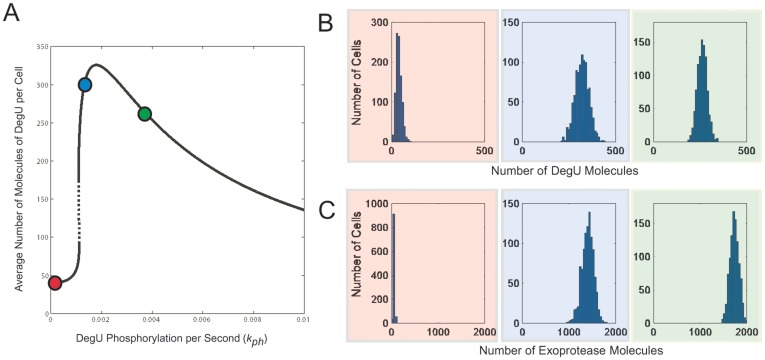
Intrinsic noise effects on steady state protein levels for different phosphorylation rates. Output from system (1)–(6) computed using the Gillespie SSA. For comparison, the DegU levels predicted by the deterministic system with the response for the chosen 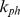 values indicated by the red, blue and green dots, respectively, are shown in (A). Histograms show the levels of: (B) DegU and (C) exoprotease collated from 1000 simulations at 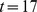 hours. All parameter values from Table 1 except in A, B and C red indicates 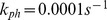; blue indicates 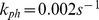 and green indicates 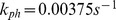. Initial values set to correspond to the equivalent deterministic steady state.

First, we considered the signal parameter 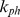, to be fixed and studied the effects of low copy numbers on the temporal evolution of the DegU and exoprotease levels. The results are shown in Figures 6 and 7. As a result of intrinsic noise, it was observed that the predicted DegU and exoprotease levels were spread across a range of values. Initially low protein level increased with time, but stabilised in less than 5 hours to distributions centred around the corresponding deterministic steady state value (Figure 6). The temporal effects discussed in [8] can be seen here: expression levels have a broad (heterogeneous) distributions at early time points. However, these distributions tighten with increasing time. These results suggest that intrinsic noise has no significant, lasting qualitative effect on system output and as predicted by the underlying deterministic system, the model predicts that a unimodal response of the cell population to a fixed environmental signal would be observed. These results were not significantly altered by using different choices of initial data (see Figure S7). Moreover, we note that this prediction is not limited to the chosen signal strength. Profiles generated using a further two values of 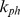 are shown at the 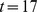 hour time point in Figure 7. These correspond to 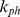 values below, at, and above the generic fold in the steady state branch. These could perhaps be considered to be low, intermediate and high levels of signal perception or transduction by DegS. In each case, the model predicted a clear unimodal response of the system.

#### Extrinsic noise: signal variation/perception

**Figure 8:**
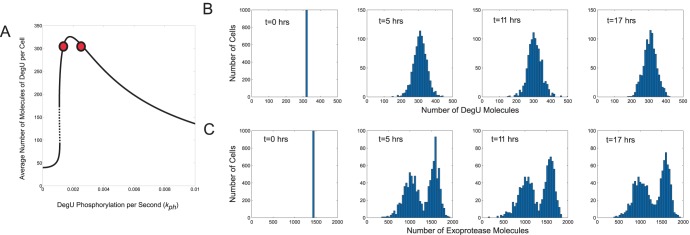
Temporal evolution of protein levels subject to extrinsic noise. Output from system (1)–(6) computed using the Gillespie SSA. For comparison, the DegU levels predicted by the deterministic system with the response for the chosen 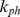 values indicated by the red dots is shown in (A). Histograms show the levels of (B) DegU and (C) exoprotease collated from 1000 simulations at 4 time points. All parameter values from Table 1 except for each simulation, 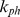 was selected randomly from the values 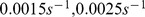. Initial values of the variables set to correspond to the deterministic steady state for 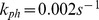.

Next, we considered extrinsic noise in the system and focussed on effects that may arise from stochastic variations in, or perception of, the input signal. These were modelled by variations in the phosphorylation rate, 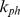. As a first step, on starting each run of the GSSA, a random assignment of two phosphorylation rates was made, the values of which represented points at each side of the generic fold as discussed above (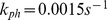 and 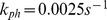). The output is shown in Figure 8. The distribution of DegU levels displayed a similar spread to that associated with intrinsic noise (cf. Figures 7B (blue) and 8B, 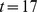 hrs). However, the spread of exoprotease levels was distinctly different (cf. Figures 7C (blue) and 8C, 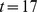 hrs). From Figure 8C, it is seen that a persistent, bimodal response is predicted. The differences in system response to intrinsic and extrinsic noise can be statistically quantified. Intrinsic noise resulted in e.g. a mean DegU level of 321 with relative standard deviation (rsd) 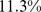 (Figure 7, blue), whereas extrinsic noise for the same system set-up resulted in a mean DegU level of 307 and rsd 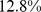 (Figure 8B at 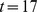
hrs). Hence, the mean and rsd remain essentially unaltered and we conclude that signal variation/perception does not significantly enhance the stochastic effects of intrinsic noise in the DegU production pathway. However, the statistics for exoprotease levels are quite different. For example, intrinsic noise resulted in a mean exoprotease level of 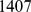 with rsd 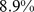 (Figure 7C, blue), whilst extrinsic noise resulted in a mean level of 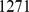 and rsd 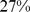 (Figure 8C at 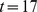
hrs). So under signal variation, the mean was slightly reduced, but most strikingly, the relative standard deviation in protease level was increased more than 3-fold.

**Figure 9:**
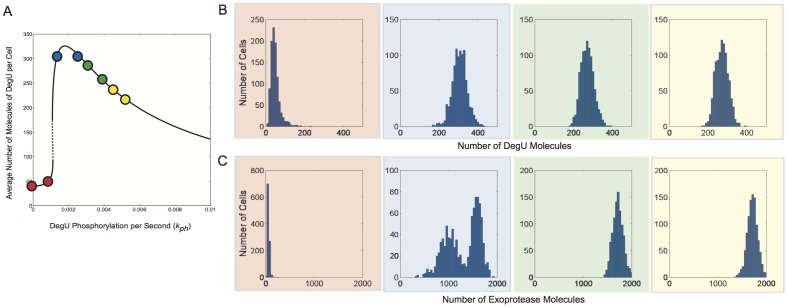
Extrinsic noise effects on steady state protein levels: perception to discrete signal changes. Output from system (1)–(6) subject to discrete steps in signal level/perception computed using the Gillespie SSA. For comparison, the DegU levels predicted by the deterministic system with the response for the chosen 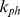 values indicated by the red, blue, green and yellow dots, respectively, are shown in (A). Histograms show the levels of (B) DegU and (C) exoprotease collated from 1000 simulations at 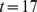 hours. All parameter values from Table 1 except for each simulation, 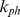 was selected randomly from the values: 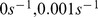 (red); 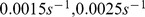 (blue); 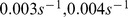 (green); 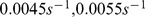 (yellow). Initial levels set to correspond to the deterministic steady state associated with the midpoint values.

We then verified that this bimodal response was not simply manufactured by selecting two different levels of 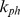. Indeed, choosing values of 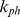 away from the region of the generic fold resulted in no predicted bimodality (Figure 9). The model predicted that the system responds in a unimodal way to a different signal input, albeit with varying degrees of heterogeneity. Moreover, for values close to the fold, the bimodality was not dependent on the initial state of the system. Figure S8 shows that bimodal system response was obtained irrespective of whether all cells were modelled as being initially all ON, all OFF or at an intermediate level of activity. These results clearly delineate this type of system response from the more typically reported bimodality that arises from a bistable switch in the underlying regulatory system.

**Figure 10:**
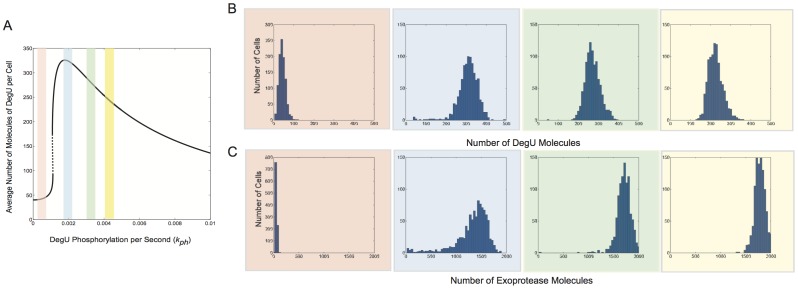
Extrinsic noise effects on steady state protein levels: perception to continuous signal changes. Output from system (1)–(6) subject to normally distributed signal level/perception computed using the Gillespie SSA. For comparison, the DegU levels predicted by the deterministic system with the response for the chosen 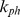 values indicated by the red, blue, green and yellow bands, respectively, are shown in (A). Histograms show the levels of (B) DegU and (C) exoprotease collated from 1000 simulations at 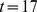 hours. All parameter values from Table 1 except for each simulation, 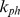 was selected from normally distributed values with mean values 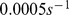 (red); 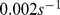 (blue); 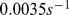 (green); 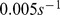 (yellow). In each case the relative standard deviation of the signal input was set at 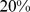. Initial levels set to correspond to the deterministic steady state associated with the mean values.

Finally, we tested the effects of selecting values of the signal parameter 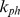 to be normally distributed about chosen values situated below, at and above the generic fold, see Figure 10. The associated statistics revealed an interesting relationship between the variance of the input signal and that of the system output, see Table 2. What is most noteworthy is that when the rsd of the signal input was increased to 20% and hence was the dominant source of noise, the system clearly responded selectively: for 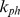 values close to the fold, the variance in DegU level matched that of the variance of the input signal, but the variance in exoprotease level was significantly amplified. Away from the fold, the variance induced by noisy signal input was in fact damped by the system. This was most pronounced for exoprotease levels. Hence, the overall sensitivity of exoprotease level to changes in signal strength was predicted to be much greater than that for DegU levels. Moreover, our analysis revealed that the nonlinear dynamics of the underlying system could either amplify or damp signal noise, depending on the level of that signal. Qualitatively similar behaviour was observed if instead, the signal was assumed to be uniformly distributed within an interval centred around the mean signal values used for Figure 10 (see Figure S9). In summary, it appears that the type of noise is not important - all that is required is signal variation.

**Table 2:** Response of System to Noise.

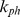 m 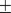 rsd	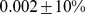		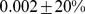		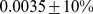		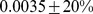	
Variable	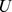	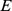	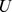	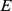	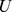	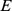	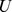	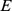
m	321	1297	307	1238	271	1717	270	1702
std	38	189	53	360	29	102	37	138
rsd	12	15	17	29	11	6	14	8

Response of System (1)–(6) to variations in the signal input 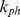. System response measured at 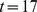
hrs. Table shows mean (m), standard deviation (std) and relative standard deviation (rsd  =  m/std*100) of 1000 simulations in each case.

### The AND Logic of the Regulatory System

**Figure 11:**
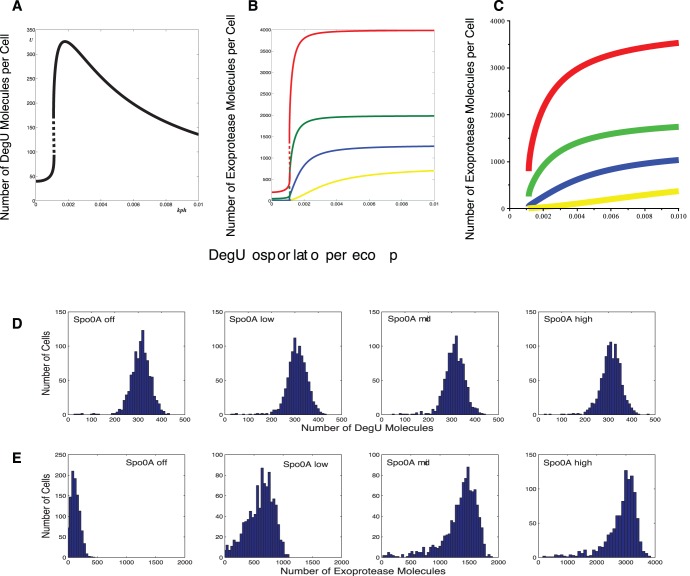
Effects of AbrB and SinR on selective heterogeneity. Effects on the steady state response of the deterministic system (1)–(6): (A) The DegU response curve is unaffected by changes in AbrB and SinR. (B) The level of exoprotease expression as predicted by the full system. (C) 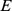 as computed from equation (S8) in the minimal system. Colours represent: Spo0A High (SinR  = 0 and AbrB  = 0) (red); Spo0A Mid (SinR  = 7 and AbrB  = 7) (green); Spo0A Low (SinR  = 14 and AbrB  = 70) (blue) and Spo0A Off (SinR  = 21 and AbrB  = 700) (yellow). (D) and (E) shows output from system (1)–(6) computed using the Gillespie SSA. Histograms show the levels of (D) DegU and (E) exoprotease collated from 1000 simulations of the stochastic model at 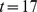 hours. All parameter values from Table 1 except for each simulation, 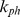 was drawn from a normally distributed set of values with mean 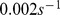 and rsd  = 20%. As above, Spo0A Off (AbrB  = 700, SinR  = 21); Spo0A Low (AbrB  = 70, SinR  = 14); Spo0A Mid (AbrB  = 7, SinR  = 7); Spo0A High (AbrB  =  SinR  = 0). Note the change of scale for exoprotease copy number in the last figure in (E). Initial values set to correspond to the deterministic steady state associated with the midpoint value.

Evidence suggests that the transcription of exoprotease requires “go” signals from both DegU and Spo0A pathways [8]. As briefly discussed above, the latter operates by inhibiting the transcription of SinR and AbrB, which in turn inhibit the transcription of exoprotease. As mentioned in the introduction, Spo0A
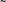
P is a transcription factor known for its role in controlling sporulation as well as exoprotease production [25]–[27]. The joint control of exoprotease production and sporulation by the one regulator presumably allows optimal survival in a dynamic environment. Sporulation is the terminal cell fate of *B. subtilis* that occurs when the levels of Spo0A
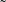
P are high [29]. As briefly discussed above, Spo0A
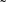
P operates during exoprotease production by inhibiting the transcription of SinR and AbrB, which in turn inhibit transcription of the exoprotease genes. This occurs at intermediate levels of Spo0A
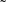
P thus prior to the activation of sporulation [29]. With this background, we assumed that changing levels of Spo0A could be modelled by changing the levels of AbrB and SinR, with AbrB and SinR levels inversely proportional to Spo0A. Figures 11A and B demonstrate the effect of varying Spo0A on the steady state signal response curves of the full system (1)–(6). The DegU expression level is unaltered. However, the exoprotease levels clearly change. As Spo0A is reduced, the exoprotease response curve flattened out (Figure 11B). Hence, the difference in exoprotease levels across the fold region shown in Figure 11A is significantly reduced. Again, we can use the minimal model to better understand the effects shown here. Briefly, from the minimal model (see equation S8 in the SI), it follows that AbrB and SinR play different roles in mediating system response. Increasing SinR decreases the maximum expression level, 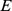, of exoprotease, whilst increasing AbrB affects the sigmoidal response of 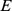 by increasing the half-maximal rate concentration of the dimer, 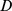, of DegU
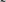
P. However, the consequence of increased levels of either AbrB or SinR is that the level of 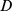 (alt. 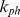) required to achieve a given level of exoprotease, is increased. In conclusion, the predicted steady state level of exoprotease plotted as a function of 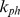 plateaus with increasing AbrB and/or SinR (Figure 11C).

Figure 11D and E illustrate the effect of increasing Spo0A on stochastic realisations of the full model. As predicted by the deterministic model, the DegU response is unaltered. However, a transit occurs from unimodal exoprotease-OFF, through a heterogenous zone, to uniformly exoprotease-ON, as Spo0A as increased. Hence, the model details why a combination of the fold in the DegU response curve *and* suitable levels of Spo0A is required to induce heterogeneity in the cell expression levels.

## Discussion

Noise in regulatory networks of the kind discussed here is clearly an important factor in determining cell fate. One common mechanism proposed for cell differentiation is the existence of an underlying bistability in the steady state structure. In this case, for a fixed system parameter, stochastic fluctuations in protein levels can result in switching between these states. Moreover, close to the deterministically defined fold bifurcations, the temporal evolution of protein levels can be significantly affected by stochastic events. This “first past the post” route to cell differentiation has been termed *transient heterogeneity* or *heterochronosity* and has been related to both protease production [8] and sporulation [50], [51]. Other mechanisms for bimodal cell distributions that do not rely on underlying bistability have been previously proposed (see e.g. [30], [52]–[54] and the references therein). However, in this paper, we introduced what we believe to be an alternative mechanism for inducing bimodality or at least *selective heterogeneity* in cell populations. This mechanism does not rely on transient effects and as such is a stable, or persistent, feature of the system. Therefore this mechanism may have relevance to understanding the development of biofilms [39]. Taking the DegU master regulatory system in *B. subtilis* as a genetically defined example, we found that a generic fold in the regulator signal-response curve coupled to a sensitive switch in the down-stream component and variations in environmental stimulus, provides an analytical framework underpinning a possible mechanism for cell differentiation. A key point is that the magnitude of variation in the signal is not in itself the driver of the selective response predicted by the model. Rather, the analysis presented here revealed that the structure of the steady state framework essentially separates system response into three zones: low stimulus levels represented by low values of a key phosphorylation rate result in low levels of DegU and exoprotease (OFF-OFF cells); there is a central zone where cells can either be DegU ON and exoprotease OFF or DegU ON and exoprotease ON; for higher stimulus levels leading to higher phosphorylation rates, the cells are uniformly ON-ON. In fact for very high levels for the phosphorylation rate, the cells remain ON for exoprotease, but reduce the level of unphosphorylated DegU (see Figure 3). We hypothesize that this last feature may be a mechanism for coordinating the down-regulation of competence whilst maintaining exoprotease production and thus represents further commitment of the population to survival strategies in response to poor environmental conditions [13]. In conclusion, we hypothesize that these zones of response represent “bet-hedging” by the system [55] – for either low or high temporally sustained stimuli, the cells have a clear and unequivocal signal to follow. The heterogeneous distribution of cells predicted for mid-levels of signal response, represents a “measured” response to a signal which may not be sufficiently well-defined to cause the population as a whole to either ignore or commit irreversibly to a certain route. This buffer region may allow cell populations to revert back to a “resting” state if conditions improve, whilst remaining prepared to respond quickly to worsening conditions.

We note that persistent, selective heterogeneity may not be restricted to the specific pathway under consideration here. The model (1)–(6) comprises generic representations of activation, biochemical interaction and degradation. As such, we believe that the fold in the steady state solution branch that forms the focus for selective heterogeneity may be a common feature of many regulatory systems that contain a bounded, positive feedback from the phosphorylated form of the protein [56].

Finally, in this paper we have highlighted differences between the transient, dynamic system response and the long-term status of a typical regulatory network. We view this as an important issue that appears to remain largely unaddressed. In a chemostat, it may be possible to hold environmental conditions essentially fixed and therefore measure cell-response to a fixed signal over what would be a *biochemical timescale*. However, in almost all natural environmental conditions and/or in the formation of complex colony structures such as biofilms, it is most likely that the status of the cell micro-environment varies in space and time. Hence, in these situations it would be difficult to separate the biochemical processes involved in cell differentiation from spatio-temporal variations in environmental signals. We believe that studying the effects of the interplay between these intra- an extra-cellular timescales may reveal new insight into the role of signal variation in determining cell fate in complex environments.

## Supporting Information

Figure S1 **Transient dynamics of DegU and Exoprotease as predicted by the full system.** The level of DegU and exoprotease as predicted by the full system (S1)-(S6). Transient response as mediated by the phosphorylation rate k_ph_ (A and B) and the initial data (C and D). All parameter values from Table 1 except (A) DegU and (B) exoprotease response for kph  =  0.001_s−1_ (black); k_ph_  =  0.0015_s−1_ (red); k_ph_  =  0.002_s−1_ (blue); and k_ph_  =  0.0025s_−1_ (green). Initial values of all reactants set to zero (OFF). In (C) DegU and (D) exoprotease, initial data chosen to represent the OFF state (0, 0, 0, 0, 0, 0) (black); intermediate state (4, 250, 10, 50, 5, 1000) (red); and ON state (5, 500, 20, 100, 20, 2000) (blue). For (C) and (D) k_ph_  =  0.002_s−1_, representing an intermediate signal level.
(EPS)

Figure S2
**Structure of system signal-response curves as predicted by the minimal system.** The solutions of the minimal system (S7)–(S9) for I_0_  =  0 as functions of the phosphorylation ratio 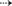. (A) DegU : U_+_ (black), U_−_ (blue) and U 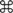 0 (red) (U_−_ + 50 is shown for ease of visualisation); (B) DegU
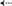
P : P_+_, (black), P_−_ (blue) and P 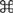 0 (red); (C) Dimer of DegU
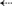
P : D_+_ (black), D_−_ (blue) and D 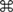 0 (red) (D) Exoprotease E_+_ (black), E_−_ (blue) and E 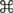 E_0_ (red) with E_0_ given by (S8) with D 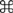 0. All other parameter values from Table 1.(EPS)

Figure S3
**DegU and exoprotesase levels as predicted by the minimal system.** The solutions of the minimal system for I_0_  =  0 as functions of the phosphorylation ratio 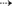. (A) DegU and (B) exoprotease. The dots represent the levels of DegU and exoprotease for values of 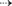 set at 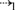
_l_ and 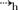
_h_, respectively. All other parameter values from Table 1.
(TIFF)

Figure S4
**Steady state signal response curves for the minimal system: e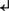 ects of increasing I_0_.** The cubic curve given in (S9) plotted as a function of the DegU level U for di
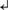
ering values of I_0_. Zeros of this cubic represent steady states of the minimal problem. (A) The general shape of the cubics over the range of values of U considered: I_0_  =  0, 10_−6_, 10_−5_, 10_−4_ (indistinguishable - red); I_0_  =  10_−3_ (blue); I_0_  =  4⇥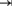 10_−3_ (black). (B) Zooming into the two small roots of this cubic. I_0_  =  0, 10_−6_, 10_−5_, 10_−4_ (indistinguishable - red); I_0_  =  10_−3_ (blue); I_0_  =  4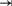 10_−3_ (black). (C) Zooming in further to the smallest root. Note that for I_0_  =  0, the cubic has a root U  =  0 whereas for I_0_ > 0 the smallest root is in fact positive. I_0_  =  0 (red), I_0_  =  10_−5_ (yellow), I_0_  =  10_−4_ (green). (D) Zooming into the largest root. I_0_  =  0, 10_−6_, 10_−5_, 10_−4_ (indistinguishable - red); I_0_  =  10_−3_ (blue); I_0_  =  4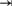 10_−3_ (black). All other parameter values from Table 1 except k_ph_  =  0.002_s−1_ 7.
(EPS)

Figure S5
**Dynamics of the minimal system.** Solutions U of (S9) with the left hand side replaced by dU/dt as functions of time. All initial values of U above approximately 11 molecules per cell result in the system tending to the steady state, U_+_ (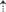 430 molecules per cell for the parameter values used here). (A) I_0_  =  10_−10_ and U(0)  =  10 (black), U(0)  =  15 (blue), U(0)  =  20 (red). (B) I_0_  =  4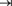 10_−3_ and U(0)  =  0 (black), U(0)  =  5 (blue), U(0)  =  10 (red). All other parameter values from Table 1 except k_ph_  =  0.002_s−1_.
(EPS)

Figure S6
**Steady state signal response curves for the full system.** Steady state solutions of system (S1)-(S6) as functions of the signal parameter k_ph_. Left columns (blue) show response for k_deph_  =  0.05_s−1_ and right columns (yellow) for k_deph_  =  0.005s_−1_. (A) From the top: k_ph_ vs DegU; k_ph_ vs DegU
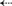
P; k_ph_ vs dimer of DegU
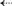
P; k_ph_ vs DegU +DegU
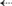
P+dimer; k_ph_ vs E. (B) DegU vs E; DegU +DegU
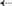
P+dimer vs E. All other parameter values from Table 1.
(EPS)

Figure S7
**E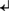 ects of initial cell profile on final system response to intrinsic noise in the signal transduction pathway.** Output from system (S1)-(S6) for three initial system configurations. Histograms show levels of DegU and exoprotease at five time points as computed using the Gillespie SSA. Data from 1000 simulations is shown in each case with output shown at t  =  0, 4, 9, 13, 17 hrs. (A) All cells initially OFF (DegU  =  E  =  0) (B) Mid initial level (DegU  =  250, E  =  1000) (C) All cells initially ON (DegU  = 500, E  =  2000). All other parameter values from Table 1 except k_ph_  =  0.002_s−1_.(EPS)

Figure S8
**E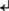 ects of initial cell profile on final system response to extrinsic noise in the signal.** Output from system (S1)-(S6) for three initial system configurations. Histograms show levels of DegU and exoprotease at four time points as computed using the Gillespie SSA. Data from 1000 simulations is shown in each case. (A) All cells initially OFF (DegU  =  E  =  0) (B) Mid initial level (DegU  =  250, E  =  1000) (C) All cells initially ON (DegU  = 500, E  =  2000). All other parameter values from Table 1 except for each simulation the value of k_ph_ was selected at random from the set {0.0015, 0.0025}.(EPS)

Figure S9
**Extrinsic noise e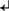 ects on steady state protein levels: uniform signal variance.** Output from system (S1)-(S6) subject to uniformly distributed signal strength/perception computed using the Gillespie SSA. Data from 1000 simulations is shown in each case. Histograms show levels of (A) DegU and (B) exoprotease at time t  =  17hrs with the signal strength parameter k_ph_ chosen uniformly from the closed intervals (i) [0, 0.001] (ii) [0.0015, 0.0025] and (iii) [0.003, 0.004]. In each case, initial values taken to represent the deterministic predicted steady state associated with the mid point of the corresponding interval. All other parameter values from Table 1.(EPS)

Information S1 **Selective Heterogeneity in Extracellular 1 Protease Production.**
(PDF)
