# Supplementary figures and images for: Selective Heterogeneity in Exoprotease Production by Bacillus subtilis
Source: PLoS One. 2012 Jun 20;7(6):e38574. doi: 10.1371/journal.pone.0038574 (PMC3380070; doi:10.1371/journal.pone.0038574)

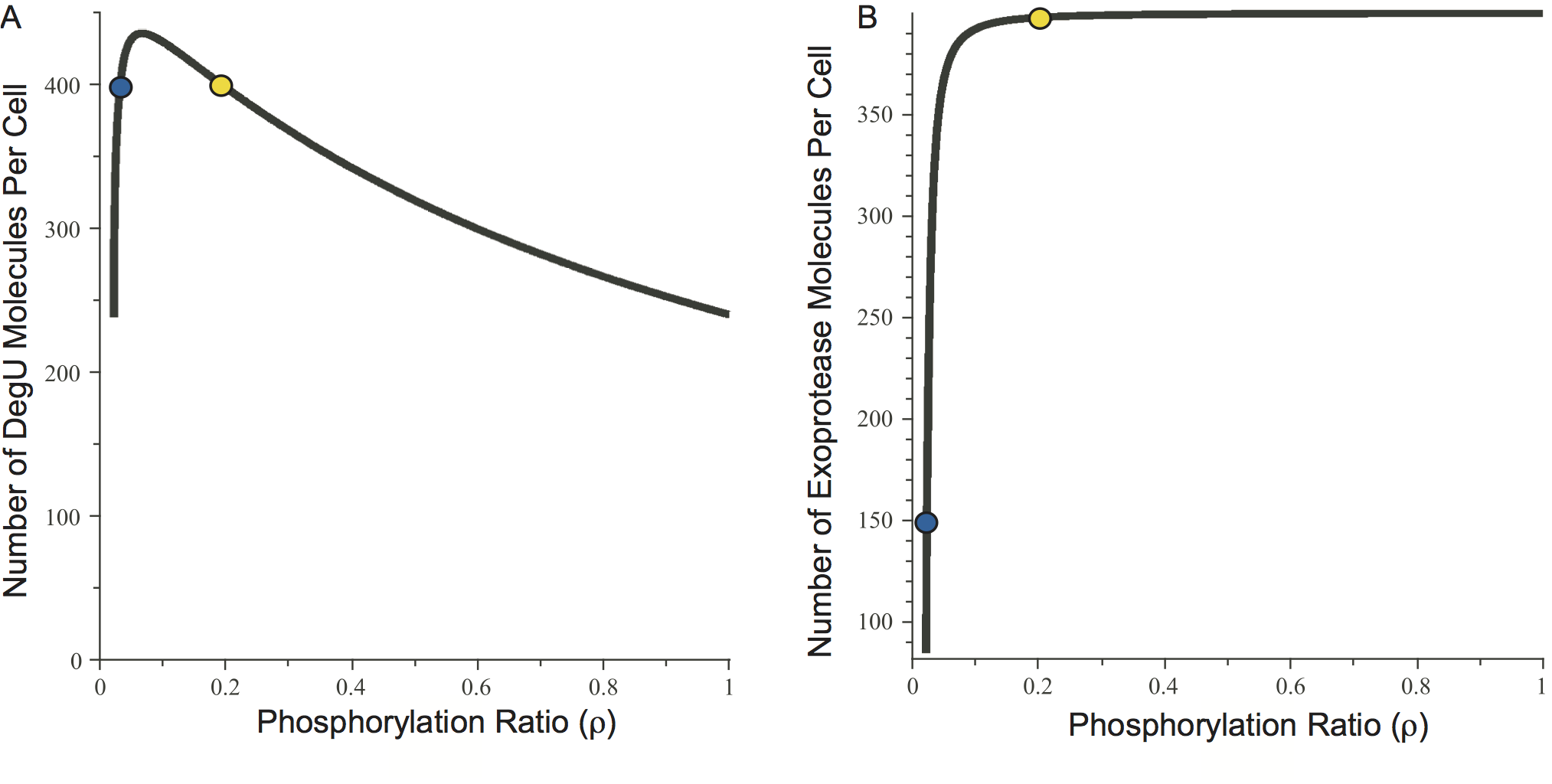

Supplement: Figure S3 — DegU and exoprotesase levels as predicted by the minimal system. The solutions of the minimal system for I0 = 0 as functions of the phosphorylation ratio . (A) DegU and (B) exoprotease. The dots represent the levels of DegU and exoprotease for values of set at l and h, respectively. All other parameter values from Table 1. (TIFF) [file pone.0038574.s003.tif]
