# Supplementary material for: Selective Heterogeneity in Exoprotease Production by Bacillus subtilis
Source: PLoS One. 2012 Jun 20;7(6):e38574. doi: 10.1371/journal.pone.0038574 (PMC3380070; doi:10.1371/journal.pone.0038574)
Supplement: Information S1 — Selective Heterogeneity in Extracellular 1 Protease Production. (PDF) [file pone.0038574.s010.pdf]

Selective Heterogeneity in Extracellular Protease Production: Supplementary Information.

Fordyce A. Davidson[1,*] Chung Seon-Yi[1], Nicola R. Stanley-Wall[2],
1 Division of Mathematics, University of Dundee, Dundee, UK.
2 Division of Molecular Microbiology, College of Life Sciences, University of Dundee, Dundee, UK.
***E-mail: fdavidson@maths.dundee.ac.uk**

1 The full system

The full system under consideration in this paper is

$$\text{mRNA DegU:} \quad \frac{dm_U}{dt} = \underbrace{\mathrm{K}_U}_{\text{transcription}} \overbrace{-k_{mdeg} m_U}^{\text{degradation}}, \tag{S1}$$

$$\text{DegU:} \quad \frac{dU}{dt} = \underbrace{k_T m_U}_{\text{translation}} \overbrace{-k_{ph} U + k_{deph} P}^{\text{phos./dephosphorylation}} -k_{pdeg} U, \tag{S2}$$

$$\text{DegU}\sim\text{P:} \quad \frac{dP}{dt} = k_{ph} U - k_{deph} P + 2 \times (\underbrace{k_d D}_{\text{disassociation}} - \overbrace{k_a P^2}^{\text{dimerisation}}) - k_{pdeg} P, \tag{S3}$$

$$\text{Dimer:} \quad \frac{dD}{dt} = k_a P^2 - k_d D - k_{pdeg} D, \tag{S4}$$

$$\text{mRNA Exoprotease:} \quad \frac{dm_E}{dt} = \underbrace{\mathrm{K}_E}_{\text{transcription}} -k_{mdeg} m_E, \tag{S5}$$

$$\text{Exoprotease:} \quad \frac{dE}{dt} = k_T m_E - k_{pdeg} E. \tag{S6}$$

The parameter values used for all simulations are given in Table 1 of the paper.

2 Dynamics of the full system: dependence on initial data and parameter values

Figure S1 shows the system response for the dynamics of the full system (S1)-(S6). In Figure S1A, the effects of setting different phosphorylation rates on the transient behaviour of the DegU component is shown. The maximum transient period is around 8 hours. The range of values of the dephosphorylation rate k_{ph} used in the figure represents values associated with the fold in the steady state response curve. This range of values returns the longest transient times: for k_{ph} outwith this range, transient times are considerably shorter. Moreover, this range of maximal transient times is insensitive even to changes by factors of 10 in all other parameters. The maximum transient time we were able to achieve by varying parameters by factors of 10 was just under 10 hours (data not shown). Note that the steady state levels are sensitive to such changes. In Figure S1B the system is initialised to be in generic low, medium and high states, respectively. Again, the maximum transient period is shown to be of the order of 8 hours for this set of parameter values.This result is insensitive to the exact quantitative form of the initial data and level of reactants. Other variables respond similarly.

3 Derivation of a minimal system

An understanding of the properties of the steady states of the full system can be gained by first making certain reasonable approximations. It can usually be reasonably assumed that phosphorylation and dephosphorylation are much faster than transcription and protein degradation and therefore when the system is close to steady state, then $P \approx \frac{k_{ph}}{k_{deph}}U$. Similarly, association and disassociation of the dimer can reasonably be assumed to be faster than protein degradation, and so $D \approx \frac{k_a}{k_d}P^2$. Finally, it follows directly from (S5) that $K_E = k_{mdeg}m_E$ in steady state. These assumptions provide a "minimal approximation" to the system (S1)-(S6) as follows:

$$P = \frac{k_{ph}}{k_{deph}}U; \quad D = \frac{k_a}{k_d}P^2 \quad \text{and} \tag{S7}$$

$$E = \frac{k_T}{k_{mdeg}k_{pdeg}}\left[\frac{I_0^E + I_{\max}^E\left(\left(D/K_D^E\right) + \left(D/K_D^E\right)^2\right)}{1 + \left(D/K_D^E\right) + \left(D/K_D^E\right)^2 + \mathrm{AbrB}/K_A}\right]\frac{K_S}{K_S + \mathrm{SinR}}. \tag{S8}$$

The variable U can therefore be determined by solving the following single equation:

$$0 = \frac{k_T}{k_{mdeg}}\left[\frac{I_0K_D + I_{\max}\frac{k_a}{k_d}\left(\frac{k_{ph}}{k_{deph}}U\right)^2}{K_D + \frac{k_a}{k_d}\left(\frac{k_{ph}}{k_{deph}}U\right)^2}\right] - k_{pdeg}U\left(1 + \frac{k_{ph}}{k_{deph}}\right). \tag{S9}$$

In [1], equation (S9) is discussed via a further simplification induced by a change of variables. The main aim of the analysis presented there was to determine the existence of a region in parameter space for which (S9) has multiple, coexisting steady states. We show here that an alternative, direct analysis of (S7)-(S9), reveals the origin of this bistability. Additionally, our approach significantly enhances the understanding of the underlying properties of the regulatory system. We will establish that the heterogenous expression of exoprotease production in a cell population does result, but not from bistability in the classical sense. Rather, it comes from a generic fold in the solution branch associated with (S9) (see Figure S2). In other words, the steady state levels of DegU and the exoproteases respond in different ways to changes in environmental signals, and more significantly, changes in these levels are differentially sensitive to the strength of the environmental signal.

4 Bifurcation and dynamics of the minimal system

The ratio of the background translation to the maximal activated rate of DegU , i.e. $I_0/I_{\max}$ can be considered small [1]. Thus, in the limit we may set this ratio to zero, or alternatively simply set $I_0 = 0$. In either case, it is straight forward to show that equation (S9) has three solutions for U, namely $U = 0$ and $U = U_\pm$ where

$$U_\pm := \frac{k_{deph}\alpha\left[k_{ph} \pm \sqrt{\beta k_{ph}^2 - \gamma k_{ph}k_{deph} - \delta k_{deph}^2}\right]}{(k_{ph} + k_{deph})k_{ph}}, \tag{S10}$$

where α to δ are combinations of other system parameters. We note that this can be neatly written in terms of a single parameter $\rho = k_{ph}/k_{deph}$, as

$$U_\pm(\rho) = \frac{\alpha\left[\rho \pm \sqrt{\beta\rho^2 - \gamma\rho - \delta}\right]}{(1+\rho)\rho}. \tag{S11}$$

Several qualitative properties of the solutions $U_\pm$ are now immediately obvious from (S11): (i) real solutions do not exist for small ρ; (ii) $U_+ = U_-$ at the critical value $\rho_c > 0$ where ρ_c satisfies $\beta\rho^2 - \gamma\rho - \delta = 0$; (iii) U_- is monotonically decreasing in ρ; (iv) U_+ is initially increasing in ρ and; (v) $U_\pm \to 0$ as $\rho \to \infty$. From these and related properties, it follows directly that U_+ has a unique maximum at some $\rho_m > \rho_c$. Figure S2A gives a schematic representation of these properties.

Using the above solutions, it follows from (S7) that $P = 0$ or $P = P_\pm$ where

$$P_\pm := \rho U_\pm = \frac{\alpha\left[\rho \pm \sqrt{\beta\rho^2 - \gamma\rho - \delta}\right]}{(1+\rho)}. \tag{S12}$$

From this expression, clearly $P_\pm$ are real and positive for $\rho > \rho_c$, $P_\pm \to \alpha(1 \pm \sqrt{\beta})$ as $\rho \to \infty$, and, an easy calculation yields,

$$\frac{dP_+}{d\rho} > 0$$

for all $\rho > \rho_c$. Similarly, $D = 0$ or $D = D_\pm$ where

$$D_\pm := \frac{k_a}{k_d}(P_\pm)^2 = \frac{k_a}{k_d}\left(\frac{\alpha\left[\rho \pm \sqrt{\beta\rho^2 - \gamma\rho - \delta}\right]}{(1+\rho)}\right)^2, \tag{S13}$$

and $D_\pm$ share the same qualitative properties as $P_\pm$. Finally, (S8) shows that the response of $E_\pm = E(D_\pm)$ is saturating on increasing the level of D, with limiting value $k_T I^E_{\max}/k_{pdeg}k_{mdeg}$. (Setting $I^E_0 = 0$ does not alter this limiting value: I^E_0 simply scales the background ($D = 0$) level.) Moreover, considering the solutions $E_\pm$ as a function of ρ, it can be shown that $E_+(\rho)$ is monotone increasing for all $\rho > \rho_c$ and using (S12), that it tends to a finite limiting value as $\rho \to \infty$ (see Figure S2B-D). Key properties of the steady states of the minimal system are (Figure S3):

- For any fixed value $U_f > U_c = U_+(\rho_c)$, there exist two values ρ_l and ρ_h, say, such that $0 < \rho_l < \rho_m < \rho_h$ and

$$\begin{aligned} &U_+(\rho_l) = U_+(\rho_h) = U_f, \quad \text{but} \\ &P_+(\rho_l) < P_+(\rho_h), \quad D_+(\rho_l) < D_+(\rho_h) \quad \text{and} \quad E_+(\rho_l) < E_+(\rho_h). \end{aligned} \tag{S14}$$

- For large ρ the signal response curve for DegU tends to zero, whilst exoprotease levels settle to a fixed high state.

Moreover, the alignment of the fold with the switch in exoprotease values can be explained as follows. The top branch of the DegU response curve, $U_+ \sim 1$ for $\rho \gtrsim \rho_c$ whereas a simple calculation reveals that $E_+ \sim (\rho - \rho_c)^2$ for $\rho \gtrsim \rho_c$. The width of the fold is $\sim \rho - \rho_c$. Hence, the switch in the E_+ solution branch always spans the fold in the DegU branch.

It is clear that if k_{ph} is allowed to vary with k_{deph} fixed, then the above arguments hold with k_{ph} replacing ρ. Therefore the distance across the fold in $U_+(k_{ph})$ at $U_f > U_c$, is given by

$$k^h_{ph} - k^l_{ph} = (c_1 - c_2)k_{deph},$$

where $c_{1,2}$ are positive constants defined by solving $U_+ = U_f$ for k_{ph}. Crucially, these constants do not depend on k_{deph}. The corresponding P values are given by

$$P_h - P_l = U_f(c_1 - c_2) \quad \text{and} \quad \frac{P_h}{P_l} = \frac{c_1}{c_2}. \tag{S15}$$

Hence, for fixed $U_f > U_c$, the distance across the fold in $U_+(k_{ph})$ is directly proportional to the choice of k_{deph} whereas both the difference and the ratio of high to low P_+ are independent of the choice of k_{deph}. From (S7), the same arguments hold for high and low D_+ defined accordingly and therefore, from (S8), for high and low E_+. In conclusion, by selecting k_{deph} sufficiently small, we have established that small differences in k_{ph} result in steady states that have the same DegU value but quite different DegU $\sim P$ values and even larger differences in the phosphorylated dimer. Therefore, the downstream effect is that small differences in k_{ph} result in large differences in exoprotease levels.

Effects of increasing $I_0 > 0$

With $I_0 > 0$, $U = 0$ is no longer a solution of (S9) and the steady states are now solutions to a non-trivial cubic equation. No useful closed form expression is available for these solutions. However, it is known that such solutions vary continuously (in the complex plane) with the coefficients and the solutions of (S9) with $I_0 \geq 0$ and those for $I_0 = 0$, namely $U = 0$ and $U = U_\pm$, are similar, see Figure S4. In particular there exist three solutions, U_j, $j = 1, 2, 3$ with $|U_1| \ll 1$, and U_2, U_3 close to U_- and U_+, respectively. The U_1 and U_2 branches in fact meet at a fold bifurcation at $k_{ph} = \infty$. As I_0 is increased from 0, this fold retreats towards $k_{ph} = 0$. Therefore, for very small values of I_0, the system has 3 possible non-trivial steady state solution for $k^c_{ph} < k_{ph} < k^f_{ph}$. On increasing I_0, the fold bifurcation at $k_{ph} = k^f_{ph}$, say, disappears when $k^f_{ph} = k^c_{ph}$. Then (S9) generates a monostable system. However, the generic fold in the upper branch $U_3 \approx U_+$ remains and hence the behaviour associated with this fold as discussed above for the $I_0 = 0$ case, still holds. For the parameter values used in the simulations in the paper, the fold bifurcation is close to the critical value k^c_{ph} and so the region for which two solutions exist for a single value of k_{ph} is very small. The end result is that the relationship between the DegU branch given by $U \approx U_+$ and the approximate exoprotease level E, is qualitatively unaltered from that shown in Figure S3.

Minimal Dynamics

Close to the steady state, the minimal system dynamics can be crudely represented replacing 0 with dU/dt on the left hand side of (S9). For $I_0 = 0$, a standard calculation reveals that U_+ is a linearly asymptotically stable equilibrium of the differential equation generated in this way. Similarly it can be shown that U_- is unstable and more easily that $U = 0$ is linearly asymptotically stable. The core dynamical system generated by (S9) is therefore (trivially) bistable for all values of $k_{ph} > k^c_{ph}$. For larger values of k_{ph}, U_- is very close to zero (Figure S2A). Thus for large k_{ph}, the system tends to the positive steady state U_+ from almost all positive initial values of U. It can be numerically verified that the stability of the steady state branches for $I_0 \approx 0$ matches that of the $I_0 = 0$ case (see Figure S5). However, once I_0 is above a very small level, a unique positive branch of steady state solutions exists that is almost identical to U_+ as defined above.

5 Steady state signal response curves for the full system

The qualitative properties of the steady state response curves manifest in the full system are robust features. Figure S6 illustrates typical steady state response curves for the full system (S1)-(S6). The generic fold in the DegU response curve is clear, with the level of DegU rapidly decreasing with increasing signal above the fold location. Moreover, the steady state response for DegU and the total DegU pool (DegU + DegU$\sim$P + dimer) is both qualitatively and quantitively similar for low to medium signal levels, k_{ph}, underpinning our focus on DegU alone in the main paper. The difference in response comes for high signal levels where the DegU level tends to zero, but the level of the total pool saturates to a positive value, as predicted in the minimal model. Finally, from the graphs of DegU against exoprotease, E, it is

clear that one value of DegU can correspond to two very different values of E (low and high, respectively), as predicted by the minimal system. Again, as the analysis of the minimal model predicts, decreasing the dephosphorylation rate k_{deph} narrows the width of the fold in the DegU signal response curve. This increases the range of DegU values which can correspond to both low and high values of E.

6 Noise in the regulatory system

In the paper, various aspects of noise in the regulatory system are considered. In particular, it was discussed that noise effects are manifest in the steady state distributions of cell types. This result differs in its emphasis from previous work where the focus was on noise effects on the transient dynamics of the underlying network. The effect of intrinsic noise was shown to be transient as illustrated in Figure 6 in the paper. This transient response was essentially independent of the initial configuration of the system as the results detailed in Figure S7 confirm. Using data that represents all cells ON, all cells OFF and cells with a range of activities did not significantly alter the transient movement of the cell distribution. Most importantly, the final, steady state distributions were essentially unaltered by changes in initial configuration.

A bimodal distribution of cell types was predicted on selecting, at random, values of the signal parameter k_{ph}. In Figure S8 we show results that demonstrate that this bimodal response is independent of the initial configuration of the system. Using data that represents all cells ON, all cells OFF and cells with a range of activities did not alter the steady state bimodal response of the system.

In Figure S9, further simulations regarding the effects of noise are shown. These complement Figures 8, 9 and 10 in the paper in that they detail further properties of extrinsic noise in the system. Figure S9 shows the effects on selective heterogeneity in the cell distribution of uniformly distributed signal values.

References

1. Veening J, Igoshin OA, Eijlander RT, Nijland R, Hamoen LW, et al (2008) Transient heterogeneity in extracellular protease production by *Bacillus subtilis*. Mol Syst Biol 4: 184.

146 # Figure Legends

Figure S1. Transient dynamics of DegU and Exoprotease as predicted by the full system. The level of DegU and exoprotease as predicted by the full system (S1)-(S6). Transient response as mediated by the phosphorylation rate k_{ph} (A and B) and the initial data (C and D). All parameter values from Table 1 except (A) DegU and (B) exoprotease response for $k_{ph} = 0.001\text{s}^{-1}$ (black); $k_{ph} = 0.0015\text{s}^{-1}$ (red); $k_{ph} = 0.002\text{s}^{-1}$ (blue); and $k_{ph} = 0.0025\text{s}^{-1}$ (green). Initial values of all reactants set to zero (OFF). In (C) DegU and (D) exoprotease, initial data chosen to represent the OFF state $(0, 0, 0, 0, 0, 0)$ (black); intermediate state $(4, 250, 10, 50, 5, 1000)$ (red); and ON state $(5, 500, 20, 100, 20, 2000)$ (blue). For (C) and (D) $k_{ph} = 0.002\text{s}^{-1}$, representing an intermediate signal level.

Figure S2. Structure of system signal-response curves as predicted by the minimal system. The solutions of the minimal system (S7)-(S9) for $I_0 = 0$ as functions of the phosphorylation ratio ρ. (A) DegU : U_+ (black), U_- (blue) and $U \equiv 0$ (red) ($U_- + 50$ is shown for ease of visualisation); (B) DegU∼P : P_+, (black), P_- (blue) and $P \equiv 0$ (red); (C) Dimer of DegU∼P : D_+ (black), D_- (blue) and $D \equiv 0$ (red) (D) Exoprotease E_+ (black), E_- (blue) and $E \equiv E_0$ (red) with E_0 given by (S8) with $D \equiv 0$. All other parameter values from Table 1.

Figure S3. DegU and exoprotesase levels as predicted by the minimal system. The solutions of the minimal system for $I_0 = 0$ as functions of the phosphorylation ratio ρ. (A) DegU and (B) exoprotease. The dots represent the levels of DegU and exoprotease for values of ρ set at ρ_l and ρ_h, respectively. All other parameter values from Table 1.

Figure S4. Steady state signal response curves for the minimal system: effects of increasing I_0. The cubic curve given in (S9) plotted as a function of the DegU level U for differing values of I_0. Zeros of this cubic represent steady states of the minimal problem. (A) The general shape of the cubics over the range of values of U considered: $I_0 = 0$, 10^{-6}, $10^{-5}, 10^{-4}$ (indistinguishable - red); $I_0 = 10^{-3}$ (blue); $I_0 = 4 \times 10^{-3}$ (black). (B) Zooming into the two small roots of this cubic. $I_0 = 0$, 10^{-6}, $10^{-5}, 10^{-4}$ (indistinguishable - red); $I_0 = 10^{-3}$ (blue); $I_0 = 4 \times 10^{-3}$ (black). (C) Zooming in further to the smallest root. Note that for $I_0 = 0$, the cubic has a root $U = 0$ whereas for $I_0 > 0$ the smallest root is in fact positive. $I_0 = 0$ (red), $I_0 = 10^{-5}$ (yellow), $I_0 = 10^{-4}$ (green). (D) Zooming into the largest root. $I_0 = 0$, 10^{-6}, $10^{-5}, 10^{-4}$ (indistinguishable - red); $I_0 = 10^{-3}$ (blue); $I_0 = 4 \times 10^{-3}$ (black). All other parameter values from Table 1 except $k_{ph} = 0.002\text{s}^{-1}$

Figure S5. Dynamics of the minimal system. Solutions U of (S9) with the left hand side replaced by dU/dt as functions of time. All initial values of U above approximately 11 molecules per cell result in the system tending to the steady state, U_+ (≈ 430 molecules per cell for the parameter values used here). (A) $I_0 = 10^{-10}$ and $U(0) = 10$ (black), $U(0) = 15$ (blue), $U(0) = 20$ (red). (B) $I_0 = 4 \times 10^{-3}$ and $U(0) = 0$ (black), $U(0) = 5$ (blue), $U(0) = 10$ (red). All other parameter values from Table 1 except $k_{ph} = 0.002\text{s}^{-1}$.

Figure S6. Steady state signal response curves for the full system. Steady state solutions of system (S1)-(S6) as functions of the signal parameter k_{ph}. Left columns (blue) show response for $k_{deph} = 0.05\text{s}^{-1}$ and right columns (yellow) for $k_{deph} = 0.005\text{s}^{-1}$. (A) From the top: k_{ph} vs DegU ; k_{ph} vs DegU$\sim$P ; k_{ph} vs dimer of DegU$\sim$P ; k_{ph} vs DegU +DegU$\sim$P +dimer ; k_{ph} vs E. (B) DegU vs E; DegU +DegU$\sim$P +dimer vs E. All other parameter values from Table 1.

Figure S7. Effects of initial cell profile on final system response to intrinsic noise in the signal transduction pathway. Output from system (S1)-(S6) for three initial system configurations. Histograms show levels of DegU and exoprotease at five time points as computed using the Gillespie SSA. Data from 1000 simulations is shown in each case with output shown at $t = 0$, 4, 9, 13, 17 hrs. (A) All cells initially OFF (DegU = $E = 0$) (B) Mid initial level (DegU = 250, $E = 1000$) (C) All cells initially ON (DegU =500, $E = 2000$). All other parameter values from Table 1 except $k_{ph} = 0.002\text{s}^{-1}$.

Figure S8. Effects of initial cell profile on final system response to extrinsic noise in the signal. Output from system (S1)-(S6) for three initial system configurations. Histograms show levels of DegU and exoprotease at four time points as computed using the Gillespie SSA. Data from 1000 simulations is shown in each case. (A) All cells initially OFF (DegU = $E = 0$) (B) Mid initial level (DegU = 250, $E = 1000$) (C) All cells initially ON (DegU =500, $E = 2000$). All other parameter values from Table 1 except for each simulation the value of k_{ph} was selected at random from the set $\{0.0015, 0.0025\}$.

Figure S9. Extrinsic noise effects on steady state protein levels: uniform signal variance. Output from system (S1)-(S6) subject to uniformly distributed signal strength/perception computed using the Gillespie SSA. Data from 1000 simulations is shown in each case. Histograms show levels of (A) DegU and (B) exoprotease at time $t = 17$hrs with the signal strength parameter k_{ph} chosen uniformly from the closed intervals (i) $[0, 0.001]$ (ii) $[0.0015, 0.0025]$ and (iii) $[0.003, 0.004]$. In each case, initial values taken to represent the deterministic predicted steady state associated with the mid point of the corresponding interval. All other parameter values from Table 1.
